# The future of milk in 2050: Dairy in the age of personalization

**DOI:** 10.3168/jdsc.2025-0859

**Published:** 2025-10-18

**Authors:** Eve Pollet

**Affiliations:** Dairy Management Inc., Rosemont, IL 60018

## Abstract

Cow milk is often considered one of the most versatile foods on the planet, with an ability to transform into multiple formats, containing thousands of bioactive compounds that serve a plethora of different purposes in the body, some of which are known, and many of which are still to be discovered. In the past 25 years, dairy has continued to be recognized as a highly nutrient-dense commodity. Advancements in the next 25 years will push culture and consumers toward enhanced demands for precision, personalization, and transparency from the products they purchase to the services they use, especially regarding food, health, and wellness. This will begin the shift for dairy and milk from a nutrient-dense commodity to an even higher value food. Dairy foods will need to align with new concepts in the future of retail and commerce that allow consumers to gain tailored solutions based on a set of criteria and personal preferences they set, with and through their personal technology. Dairy science will play a critical role in enabling this shift—encompassing fields from nutrition science and product development to genetics, marketing, and commercialization—by leveraging the versatility of cow milk and demonstrating dairy food's health and wellness benefits from the molecular level to the whole dairy matrix.

## Highlights

• In the near future, customization and personalization may help fill knowledge gaps around nutrition, providing consumers with accessible and credible information based on their own biological data.
• Dairy's wide array of bioactives and macro- and micronutrients makes it a powerful, nutrient-dense product for a future in which health and wellness solutions are increasingly tailored to individual needs.
• The dairy industry should seek to leverage technology to provide consumers with the science-backed products that serve a variety of personal health and wellness needs.

## Graphical Abstract

**Figure:**
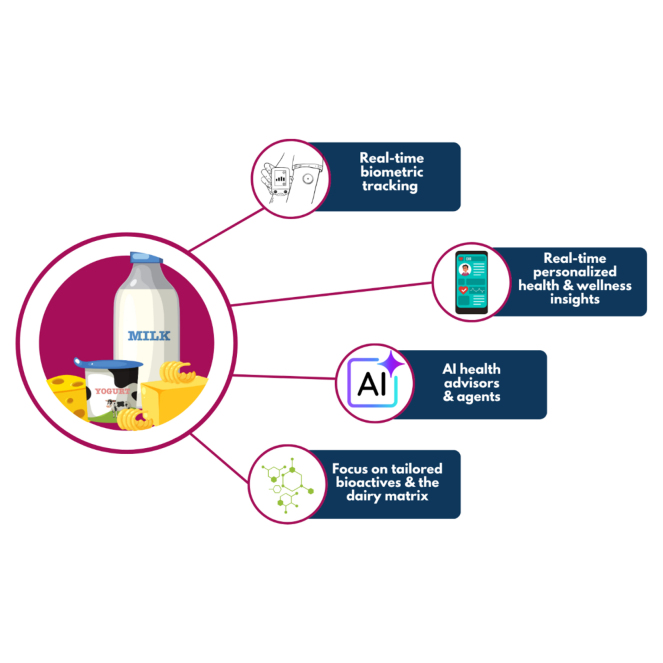
**Summary:** In the past 25 years, dairy has continued to be recognized as a highly nutrient-dense commodity. Advancements in the next 25 years will push culture and consumers toward enhanced demands for precision, personalization, and transparency—from the products they purchase to the services they use—especially regarding food, health, and wellness. This may begin the shift for dairy and milk from a nutrient-dense commodity to an even higher-value food. Dairy foods may need to align with new concepts in the future of retail and commerce that allow consumers to gain tailored solutions based on criteria and personal preferences they set, with and through their own technology. Dairy science will play a critical role in enabling this shift—encompassing fields from nutrition science and product development to genetics, marketing, and commercialization—by leveraging the versatility of milk and demonstrating dairy's health and wellness benefits from the molecular level to the whole dairy matrix. Graphical abstract created in Canva Pro.

Personalization and customization will become a driving force for change in the future, enabled by technology and affecting every industry. This article will review how technology is driving future consumer and cultural expectations toward receiving personalized recommendations, products, and services in the next 25 years. This article will examine how personalization will affect health and wellness, food and beverage, and retail. It reviews technologies, companies, and concepts that exist today that are driving this future such as those in precision health and nutrition, and how dairy science could play a critical role in this shift.

Today's average consumer under 40 yr of age has grown up in an era of constant technological advancement, experiencing important upgrades in consumer technology nearly every year. This single truth has shaped their lives, expectations, and the role of brands and food and beverage in their lives. Using a single example, since 2011, Apple has consistently released a new iPhone every year (Grothaus, 2025), setting a new expectation for consumers and culture across all categories beyond their technology: that quality upgrades are a new norm, and as a result, upgrades are a constant expectation for brands and categories. This has certainly translated to food and beverage like it has in other categories. This concept of the consistent upgrade as a norm and expectation plays out in consumer trends, consumer expectations, and behaviors. Today, novelty is the norm. Convenience and constant connection and access to the internet, to information, and to others is a baseline expectation for consumers today. As a result, attention has become the new currency in branding and business for quite some time, driven by the constant evolution of technology and digital tools (Davenport and Beck, 2002). Consumers expect upgrades from their brands, new and novel ways of enjoying them, and new features, and more recently, they have come to expect products and services to better serve their precise and contextual needs and desires. Whereas today novelty is a norm and convenience and constant connection is a baseline expectation, as we make our way to 2050, the new consumer and cultural expectation will be around personalization and customization. Today, consumers are getting personalized recommendations based on their past purchases and current needs and preferences, whether it is a targeted advertisement on their social media to a recommendation from a retailer like Amazon, or a search on Perplexity AI on what they should eat or do that returns results based on their stored personal preferences. The ability for technology to personalize product recommendations to consumers, and the ability for consumers to customize a product or experience to their specific needs and preferences will not only become a baseline expectation in the future, but it will also become a differentiator for industries, companies, and brands. Today, when consumers lack complete knowledge on products or solutions that meet their specific needs, they often give their attention to their friends, social media, and related forums or influencers to help them find solutions. This can lead to misinformation, especially when it comes to food, beverages, and supplements. A 2024 consumer survey by company MyFitnessPal and a follow-up study with MyFitnessPal and Dublin City University found that only 2.1% of nutrition trends shared on the social media site TikTok provided accurate information when compared with public health and nutrition guidelines (Dublin City University and MyFitnessPal, 2024). The survey also highlighted that 87% of Millennial and Generation Z TikTok users have turned to the platform for nutrition and health advice. For those who have been influenced by nutrition and health trends on TikTok, 67% report they adopt at least one of these trends, sometimes called “hacks,” a few times a week (Dublin City University and MyFitnessPal, 2024).

In the next 25 years, customization and personalization will help to fill knowledge gaps around nutrition, ensuring that consumers do not need to “hack” their way to solutions, by simply providing the right solutions that already exist—recommendations designed directly from the consumers' health and personal data sources, or their personal “source of truth.” New “personalized sources of truth” will have an impact on all industries, including food and beverage. They may drive new design of foods, supplements, or formats that fit consumer needs and push for a more agile infrastructure to service personalized products, from packaging to product delivery, and to product contents. Or, they may simply outline what seems to be customers' top areas of need, which require more evolved products to serve them adequately.

Although personalization will affect every area of life in the future, its impact on the health and nutrition may be one of the most exciting areas for dairy science in the future. Constant access to personalized recommendations on what consumers should eat, whether from an artificial intelligence (**AI**)-driven tool designing a meal plan based on personal preferences or from companies that allow consumers to measure a variety of biomarkers to provide insights on health and nutrition, will become the norm in the next 25 years. The personalized nutrition, wearables, and AI-powered nutrition markets are set to grow markedly in the future. The market for AI in personalized nutrition is growing by more than 23% annually across the globe, with North America as the market leader as of 2023 (The Business Research Company, 2025). About 41% of Americans now own a smartwatch or fitness tracker, and about 4 in 10 health app users track their diet with these tools (Vaidya, 2023). Devices that can track sleep, activity, heart rate, and more are affecting the next generation of consumers, who may look to technology over their doctor for health advice, protocols, and recommendations. For example, wearables like the Fitbit Ace, designed for children 7 yr of age and older, attempt to gamify the wearable experience to help kids get more activity by providing personalized information and ways to get that activity. Technology will advance with many ways to track health—from wearables to biomarkers gathered from samples, DNA data, device data, behavioral, contextual, and environmental data, and more—to provide a real-time picture of a consumer's health status. It can be speculated that in the future, a phone, wearable, or AI advisor may collect more data and know more about a consumer's body in a day than a doctor could learn in their grandparent's lifetime, due to the amount of data that can be collected by technology on a constant basis.

Personalized nutrition companies such as InsideTracker and ZOE focus on collecting biomarkers directly from consumers' biological samples and outside data such as those from wearables, allowing the companies to assess their consumers' health in a variety of areas and then provide nutrition and lifestyle recommendations to their customers via an application. To date, InsideTracker collects blood samples from consumers, integrates their wearable (if they choose) and genetic data, and provides nutrition and lifestyle recommendations for consumers to improve in a health area of their choice (https://www.insidetracker.com/). Consumers receive “scores” for specific health areas and then choose the areas they would like to work on. ZOE collects blood and stool samples along with continuous glucose monitoring (**CGM**) data from CGM wearables and consumer-supplied nutritional data to provide personalized metabolic analysis and individualized nutrition plans (https://zoe.com/en-us). InsideTracker and ZOE are shaping the future of health recommendations by allowing consumers to bypass a doctor or nutritionist and go directly to diagnostic testing and recommendations with a company. It is important to note that in many cases, both InsideTracker and ZOE are recommending dairy products, such as fermented dairy to cheese and milk, in their personalized recommendations. In addition to startup companies such as InsideTracker and ZOE, the most influential technology companies in the world are also shaping this future where the consumer has increased oversight and insight into their health. Apple has been public about making large moves into health. The patents filed by Apple reflect a potential to provide personalized health diagnostics and recommendations. Some of their devices can double as healthcare devices. Apple watch's Atrial Fibrillation History feature gained FDA approval as a Medical Device Development Tool (FDA, 2023). Rumors of Apple's Project Mulberry, a project to bring an AI advisor to their Apple Health app and Apple watch next year, were reported on this year. Publications like Bloomberg's *Power On* newsletter and *Forbes* reported on Project Mulberry and the potential for an Apple “AI doctor” to analyze biometric data to give personalized recommendations on nutrition, lifestyle, and health (Gurman, 2025). As one can imagine, with the influence Apple has had on culture in the United States and around the world, once these features become available, they could become a baseline expectation for consumers and culture and shape new norms by 2050. Samsung (https://www.samsung.com/us/apps/samsung-health/) and Google (https://store.google.com/product/pixel_watch_2?hl=en-US) have also provided health tracking and recommendation features through their wearables and devices, and they may develop similar features.

While today, in 2025, early adopter consumers are using personalized nutrition services that gather biological samples and integrate other biological data, technology costs will be reduced by 2050, and these concepts may continue to scale as consumers experience the value. Recently, as this field has expanded and grown, multiple studies have shown how precision nutrition has led to better health outcomes (de Toro-Martín et al., 2017). These companies can conduct n of 1 “trials” in real time, allowing consumers to see the cause and effect of multiple food and lifestyle decisions. One example where this is gaining attention is through the adoption of continuous glucose monitors by everyday consumers, not just people with diabetes. One woman, a former biochemist and now social media influencer, Jesse Inchauspé, also known as the Glucose Goddess on Instagram (https://www.instagram.com/glucosegoddess/?hl=en), has formed a large community of followers, with 5.8 million followers on Instagram alone as of October 2025, by posting continuous glucose monitor results from herself or others in her community, along with nutrition and lifestyle tips. The results she posts compare different common foods, food combinations, and even food timing and how they can affect glucose spikes. She compares different types of plant-based and dairy milks, different types of dairy products with different fat levels, and even the same foods but eaten at different points in time, or during a menstrual cycle. Where it gets interesting is where 2 people eat the same food and one sees a larger glucose spike. This speaks to the gaps in knowledge around individual differences, unseen factors and environmental factors at play, and the growing ability for consumers to grasp that “one size does not fit all” when it comes to healthcare or nutrition. As more consumers can track the cause and effect of their foods through continuous measurement, they may change their daily habits with real-time insights as they notice patterns. This will affect the food industry, because consumers will be able to see the effects of each product they use. This will expose what is working and what is not for consumers. It has been rumored that Apple and Samsung are working on noninvasive blood glucose monitoring features for their wearables, with researchers exploring noninvasive CGM options (Williams, 2025). If these features come to fruition and adoption of CGM becomes even more widespread, it may force food and beverage companies to confront consumer results in real time and become more agile in the product development process. Innovation cycles may need to become quicker and more responsive, like the food and beverage industry's response to GLP-1. Danone's Oikos Fusion line, a response to GLP-1 users' specific needs, shows how the industry can respond to more specific and contextual consumer needs (DiNapoli, 2025). As consumers see the value of personalized nutrition and drive its adoption, it is important to note that the timeline, complexity, and ability of the food and beverage industry to respond is still in question. However, companies may consider learning from these concepts early on. They may consider starting the research and development process incorporating results from these technology platforms in addition to real-time data from consumers that incorporate biomarkers, preferences, and more.

How dairy currently fits into a future of precision nutrition is exciting, given that companies like ZOE and InsideTracker recommend dairy foods to their users from their data. When it comes to more agile and responsive innovation cycles, with ZOE, we see the direct pipeline to product development as ZOE launched their first kefir-based “Gut Shot” in partnership with UK retailer Marks and Spencer with 14 different bacterial strains, berries, and a kefir base (https://www.marksandspencer.com/food/the-gut-shot/p/fdp60646830). The product creation drew on ZOE's research and data from their users along with the PREDICT program study that enabled ZOE researchers to quantify and predict individual variations in postprandial responses to standardized meals in addition to gathering lifestyle information (Berry et al., 2020). ZOE choosing dairy as their first food and beverage product is a signal that the future is bright for dairy within precision nutrition systems.

As more consumers begin using personalized health and wellness technologies, their health results and behaviors may become a new platform for consumer socialization. Sharing a “sleep score” or “step count” with families and friends has been a feature for some time in wearable technology such as the Oura Ring. Consumers and families have friendly competitions over their sleep scores or step counts, motivating each other to be healthier. Precision nutrition and wearable companies have recognized this and created communities around sharing tips and tricks by creating messaging boards and platforms where people can share and seek advice. In the next 25 years, with more adoption of these technologies, and as consumers become more fluent in assessing results and “speaking the language” of their biometric data, we may see more of this. We may see a new way of interacting and socializing and a new class of influencers emerge from these platforms. Today's social media influencers generally share tips and tricks and let followers in on the intimate details of their lives, gaining trust and influence as a result. Looking out to 2050, we may find that nothing is as intimate or trustworthy as sharing the data coming directly from your body, with the biometrics and the tips and tricks that come with it. Consumers may find that they are more similar than different, in some cases, as they share and socialize around their health insights, and convene around a set of guiding principles on how to live, eat, and move. Consumers may find others like them and discover that their preferred influencer might be someone who shares their biological similarities and health goals, or has achieved the health outcome they desire. The new social media and connection in the next 25 years may be more biologically driven and centered around health optimization. This represents an opportunity for the products and solutions that generally do good for consumers in this era of tracking.

The new influencers may “look just like you”—but at a biological level. Or, they may actually “be you”—your digital twin, that is, your future AI health advisor or doctor. Digital twin technology, which involves virtual replicas of a person, project, object, or system, is set to upend the future of business and the future of healthcare and nutrition (Halamka, 2024). The ability to create a digital twin and simulate outcomes and solutions before implementing them in the real world has incredible implications for health, retail, and commerce, and all aspects of life and business. In March of 2024, Chinese president Xi Jinping announced that digital twin technology was one of “six new productive forces” in which China will lead in advancement ahead of the United States (Laubenbacher, 2024). The ability of digital twin technology to take your personal data and build a virtual version of you to simulate effects of products or recommend them cannot be underestimated when it comes to health and nutrition and will likely guide consumer decision making at a level we have not seen. For example, when eating at a restaurant, consumers could scan the menu or take a photo of a food, and have their digital twin simulate the health effects from each dish based on their biometrics that day before they decide what to order. This visual prediction technology is being developed by researchers such as those at the Artificial Intelligence Institute for Food Systems at the University of California–Davis (https://aifs.ucdavis.edu/research/projects/prediction-of-health-effects-from-food-photos).

From this technology, consumer expectations and behavior will certainly shift by 2050. A growing concept in culture and the scientific community around “food is medicine” and using food as a tool to treat or prevent disease would certainly become more prominent in the future. With new precise technological tools in this future, consumers may learn insights about their own individual health that are contrary to what they previously thought. For example, as AI, science, and technology advance and build upon each other, we are seeing certain myths “busted” and new discoveries around cures for unexplainable health conditions. For example, the cover of *Scientific American*'s June 2025 issue was titled “The Sunshine Cure” about how UV light and phototherapy can become a cure for autoimmune disease. In the article, Jacobsen (2025) notes that autoimmune diseases affect about 350 million people worldwide, and several medical studies hint that UV therapy may work for a handful of conditions such as type 1 diabetes, rheumatoid arthritis, Crohn's disease, and colitis. This is a paradigm shift from the advice most consumers are getting today, especially from their dermatologists, which is largely to block the sun's rays and wear broad-spectrum sunscreen every day (American Academy of Dermatology Association, 2025). As science advances and more is understood about sun exposure and its impact on human biology, in 25 years, consistent sun exposure may be recognized as a driver of good health. The same paradigm shifts may happen in food and beverage as these technologies scale. For example, many consumers are told to follow food elimination diets or remove dairy when they have unexplainable conditions such as autoimmune disease. When science continues to advance as AI is applied to multiple scientific fields to uncover new insights, and as consumers have the technologies to measure their health at their fingertips, we may find certain foods are not a culprit, but a solution, or that food may not play as much of a role in certain conditions as we thought.

As consumers can measure effects of foods in real time and get targeted recommendations from digital twins, AI advisors, and more, they may also come to expect more targeted outcomes from their food as a result. They may expect foods to deliver on the health outcomes they are looking for more precisely in real time, or the health areas they are looking to improve. Currently, we are seeing more customized products in the marketplace, from supplements to functional foods and beverages that claim to provide a variety of targeted health benefits from immunity to sleep, stress relief, gut health, mental cognition and focus, and more. One company, Gainful (https://www.gainful.com/), currently does this by having consumers take a survey on their websiteto outline what benefits they are seeking, and then Gainful recommends products from their portfolio, such as different types of whey protein powder ingredient blends for specific desired health outcomes. This represents an opportunity for dairy science to advance to meet the consumer where they are in 2050, in whatever targeted nutritional recommendations they are getting. Dairy milk's wide array of bioactive compounds, along with its macro- and micronutrients, make it a powerful, nutrient-dense platform for a future where health and wellness are increasingly tailored to individual needs. However, this requires an even deeper understanding of what precisely makes dairy foods beneficial. The concept of the food matrix has emerged and grown in the past decade showing how the health effects of foods are based on the structure and composition of whole foods. Although much is known about the vitamins, minerals, and macronutrient composition of dairy foods, our understanding of the other unique bioactive compounds in dairy continues to evolve. Improved accessibility of standard techniques coupled with emerging technologies will help accelerate the identification and study of bioactive compounds from dairy products. Researchers are currently seeking a fuller understanding of what is in food, by looking deeper into what makes foods healthy at a molecular level. David Wishart at the University of Alberta is one of the early pioneers who attempted this with the Milk Composition Database (https://mcdb.ca/about), which looks at metabolites in milk, in addition to the FooDB (https://foodb.ca/), which Wishart laboratory claims is “the world's largest and most comprehensive resource on food constituents, chemistry and biology.” This database includes information on over 26,500 food compounds and associations including flavor, color, texture, taste, and aroma. The Rockefeller Foundation's Periodic Table of Food Initiative is preparing for this future in 2050, where nutrition is personalized and preventative, and consumers can get nutritional recommendations because molecular datasets on the composition of foods exist. Their near-term goal, as stated, is “filling the black hole of nutritional knowledge by uncovering the biomolecular composition of foods” at a global level starting with about 1,650 global foods, and this number may expand in future years (Hamilton, 2024). Private companies such as PIPA (https://pipacorp.com/) are using AI to assist private companies with the discovery of bioactives for commercialization. These efforts and the research of other scientists around the world specifically to understand the components in dairy, how they work, and how they work together, will unlock a new generation of innovation for dairy and, hopefully, health for consumers. The industry has seen great success to date in studying, commercializing, and uncovering beneficial components such as lactoferrin, milk fat globule membrane, and more. Studying and learning more about the matrix and its components will be a prerequisite for unlocking the unique value proposition that is dairy milk and dairy foods. The more funding directed toward uncovering what is in dairy and what dairy can do, the more dairy can move into 2050 successfully as a broad-spectrum health solution.

In addition to AI doctors, consumers will also get personalized product recommendations in general, beyond health and wellness, from AI agents. In addition to digital twins, the rise of agentic AI and recent programs announced by Mastercard and Visa, for example, point to a future where AI agents handle the full end-to-end process of shopping, discovering new products, providing recommendations, negotiating prices, and purchasing products or services on a consumer's behalf by learning consumers' personal behaviors and preferences (Constantino, 2025). Whereas 25 years ago it would have been unimaginable to order shoes on a cellphone from the couch to be delivered at home the next day, similar concepts may be said of agentic AI in the next 25 years, in that the product discovery process we know today may become obsolete. The connection of multiple platforms or systems that understand consumers' data will unlock the new products and services that they are looking for. They will also navigate through the noise of advertising by scanning honest reviews and price comparisons. Beyond personalized agent recommendations, consumers will of course have “of the moment” desires to participate in cultural trends or moments in the marketplace. Consumers may be able to shop their favorite TV shows or entertainment, as with Amazon's recent announcement of a “Shop the Show” feature that allows viewers to immediately shop for items that are displayed on a TV show (Ludmir, 2025). This, along with social media–enabled shopping, will provide seamless integrations of shopping in a specific cultural moment. It must be noted that the future of consumption for dairy foods relies on cultural relevance and dairy food's ability to be included in the retail concepts of the future, in addition to becoming a part of the future of personalized health and nutrition.

New advancements in AI and the ability to understand and analyze larger datasets to identify real-time personal consumer needs, preferences, and more by 2050 will push for a new infrastructure for commerce that is more direct-to-consumer or direct to their future AI agent or doctor, than ever before. It will demand a new infrastructure for research and development and innovation within the food and beverage industry in the United States in addition to a new way of marketing and commercializing goods, including dairy. Consumers not only will demand products that serve their precise needs but also satisfy their desire to participate in cultural moments of fun and enjoyment and for product features. Their agents will be able to shop for a variety of factors such as their health data, their entertainment, cultural, or social preferences, and their desire to be a more “conscious” consumer. With new technologies and advancements, consumers may develop new desires and preferences. For example, as consumers get more precise information on their health, they may be concerned not only about what is in the product but what the product is in, and how product packaging affects health. In the past couple of years, there have been multiple media reports on the health impacts of plastic and the abundance of microplastics in the water supply and environment, blood samples of consumers, and food and beverage products (Carrington, 2022). More science and technologies will enable transparency and reveal the positives or negatives about a variety of product features, including nutrition and packaging. The dairy industry may be driven to reimagine how dairy foods are a sustainable health and wellness solution. With dairy foods being versatile, packaging will be one example where dairy foods can meet the immediate needs of consumers. Currently, there are great science projects being conducted on the ability to transform dairy byproducts, such as lactose, into biodegradable plastics, such as Ruihong Zhang's initiative at the University of California–Davis (Gautam, 2023). This showcases how dairy foods can be truly circular from farm to consumer. The dairy industry can aim to turn its waste streams into “gold,” as it has historically done with whey protein (Draper, 2025). In doing so, dairy can align with the plethora of product features or criteria that consumers, or their AI agents, will look for in the future.

Although the exact timeline is unknown for when personalized nutrition, personalized AI systems, and AI agents will deeply affect and drive substantive shifts in retail and food and beverage, it will certainly reveal a new layer of complexity for infrastructure to meet the personalized needs for consumers in the future. In addition, investment demands might challenge the ability for this future to become realized quickly, especially within the dairy industry. Research and development may need to become faster and more flexible, using AI to centralize and gain knowledge about consumer health insights, dairy science, and processing innovations for speedier innovation cycles, with a more flexible pilot plant infrastructure that allows for more rapid innovation to meet consumer needs in real time. In addition to having the infrastructure in place to more quickly respond to consumer needs, uncovering what is in milk and dairy products at the molecular level is certainly required for a future in which consumers are looking for tailored health solutions. This has broader implications for the full dairy-value chain, from genetics to feed on the farm, which affect certain milk components that consumers may value in the future, to processing techniques that preserve certain bioactives, and new product formulations and formats that may be required.

Cow milk has been a versatile platform for millennia. As we are now moving closer to 2050 than to 2000, the ability of dairy to transform into a solution that can fit in to precise consumer needs is well positioned to grow over time. So much will be discovered around health and food in the next decade. To capture this value will require the efforts of the dairy science community and its funders to unlock the “magic” of the dairy matrix by discovering the unique bioactives that make dairy an incredible health food. This will reposition dairy as a high-value food uniquely capable of meeting consumer needs and supporting targeted health outcomes. It will require a greater agility and investment into research and development and the innovation process by dairy companies. It will require the ability for companies to incorporate learnings from technology and data into their research and development process to meet the precise health needs of consumers in the future, combined with continued efforts to improve product contents and packaging, upcycle waste streams for greater value, and more.
